# Neutrophil extracellular traps in cancer: immune modulation, therapy resistance, and the dilemma of targeting

**DOI:** 10.1038/s41419-025-08218-3

**Published:** 2025-12-08

**Authors:** Marta Brambilla, Anna Zanichelli, Valeria Cancila, Mario Paolo Colombo, Claudia Chiodoni, Sabina Sangaletti

**Affiliations:** 1 Molecular Immunology Unit, Department of Experimental Oncology, Fondazione IRCCS Istituto Nazionale dei Tumori, Milan, Italy; 2 Department of Medical Oncology, Fondazione IRCCS Istituto Nazionale dei Tumori, Milan, Italy; 3 Tumor Immunology Unit, Department of Sciences for Health Promotion and Mother-Child Care “G. D’Alessandro”, University of Palermo, Palermo, Italy

**Keywords:** Innate immunity, Cancer microenvironment

## Abstract

The release of extracellular DNA is a highly conserved mechanism across species. Extracellular traps released by neutrophils (NETs) represent a strategic defensive tool of the organism against pathogens, through their direct entrapment and killing, but also through the immunoadjuvant ability of their components. In this review, we discuss the involvement of NETs in cancer, from tumour initiation to recurrence, touching also on their role in cancer-associated comorbidities. We also discuss the emerging contribution of NETs in resistance to chemotherapy, radiotherapy and, particularly, immunotherapy, because of their suppression of cytotoxic immune responses and remodelling of the tumour microenvironment into an immune-tolerant niche. The therapeutic dilemma lies in whether targeting NETs to restore anti-tumour immunity may compromise the host’s defence against infections, an adverse effect that is far from negligible in patients who are often already immunocompromised. Furthermore, despite their widely recognized pro-tumoral functions, NETs have also been implicated in supporting anti-tumour immunity in specific contexts. The key challenge, therefore, lies in distinguishing between “bad” pro-tumoral NETs and “good” anti-microbial/anti-tumoral NETs, both for identifying reliable biomarkers and for developing more precise NET-based therapeutic targets.

## Facts

- **NETs play dual roles**, supporting tumour progression while also contributing to antimicrobial defence and potentially enhancing anti-tumour immunity
- The detection of extracellular traps in the TME suggests **alternative sources or subtypes of NETs** that may escape normal clearance mechanisms associated to dying neutrophils
- **Redirecting NETs functions** could enhance immunotherapy responses by overcoming resistance and restoring immune sensitivity.
- Selective targeting of **NETs** is needed to block harmful effects without losing protection

## Introduction

### Extracellular DNA extrusion: an evolutionary conserved mechanism

**Fig. 1:**
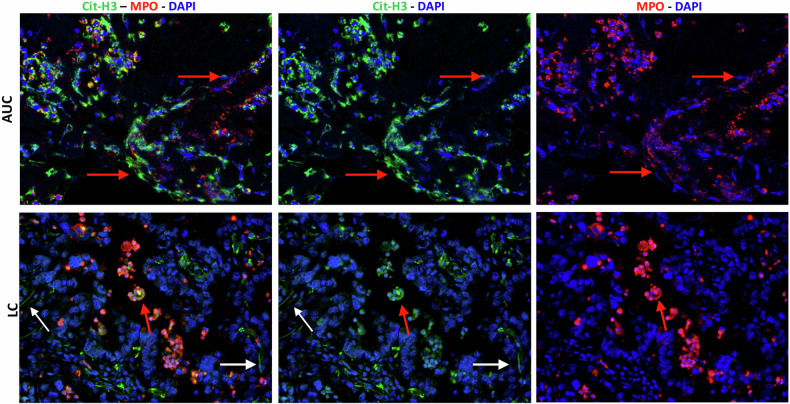
MPO-negative (-) NETs are detectable in TMA sections from lung cancer (LC) patients. Representative images of NET detection in tissue biopsies from an autoimmune condition (AUC) and a lung cancer (LC) patient. Red arrows indicate citrullinated histone H3^+^ / MPO^+^ NETs; white arrows indicate citrullinated histone H3^+^ / MPO^−^ NETs.

Neutrophil extracellular traps (NETs) consist of double-stranded DNA -chromatin structures decorated with histones and anti-microbial proteins extruded by neutrophils with the function to entrap and kill bacteria [1]. The discovery of NETs brought to light the important concept that chromatin may have evolved not only with a role in the perpetuation of life, but also by incorporating an immune and protective function [2]. Indeed, owing to the fundamental role in host defence, the release of extracellular DNA has been documented across different and distant taxonomic groups, being present in protozoans, bacteria, plants, invertebrates, fishes, and mammals. However, the extrusion of traps was of particular significance for multicellular organisms to safeguard the perpetuation of life and protect from infection. This has led to the evolution of sophisticated defence mechanisms that include the suicide of the cell termed NETosis, which was commissioned to a specialized and terminally-differentiated subset of immune cells, namely neutrophils [3]. Zhu and colleagues have recently demonstrated why NETosis could occur regardless of infection, not as a suicide but as a second type of cell death. They demonstrated that when neutrophils are triggered by a pro-apoptotic stimulus (or by any other triggers of neutrophil death) without being swiftly removed (e.g. by scavenging macrophages), the activation of membrane pore-forming enzyme (e.g. GSDME), promotes calcium influx with the consequent activation of PAD4 that citrullinates histone H3 [4], an epigenetic modification which is mandatory for NET extrusion[5]. Overall, this explains why there is a huge amount of NETs in biopsies from autoimmune patients, for which a defective clearance of neutrophils has been reported to be part of the pathogenic mechanism leading to autoimmunity [6]. This is consistent with our recent publication, in which we demonstrated that *Sparc*-deficient PMN, by escaping macrophage-mediated clearance, become a source of autoantigens, also through NET formation, in a rheumatoid arthritis model [7]. Whether a defective ability of macrophages to clear dying neutrophils, as well as whether potential defects in the ‘eat-me’ and ‘don’t-eat-me’ signals in neutrophils are involved in cancer-associated NETosis is unclear. The association between NETosis and impaired macrophage clearance of dying neutrophils raises important questions about the presence of NETs in the tumour microenvironment, which is typically enriched in M2-like scavenger macrophages. The presence of NETs in the tumour microenvironment (TME) may, at least partially, be attributed to distinct sources or subtypes of NETs. Figure 1 illustrates representative examples of NETosis observed in a biopsy from an autoimmune SLE (AUC) patient and in a biopsy from a patient with NSCLC. In biopsies from autoimmune patients, nearly all NET threads are positive for both citrullinated histone H3 (H3Cit) and myeloperoxidase (MPO) (Fig. 1, red arrows). In contrast, within the TME, H3Cit⁺/MPO⁺ NETs (red arrows) coexist with H3⁺/MPO⁻ structures (Fig. 1, white arrows), the latter displaying a more thread-like morphology. This observation supports the existence of distinct sources or subtypes of extracellular traps within the TME.

### Extracellular trap extrusion from vital neutrophils

Not all kinds of extracellular DNA extrusion imply cell death. At least two distinct mechanisms have been described for vital NET formation: 1. the release of mitochondrial DNA (mtDNA) and 2. the extrusion of nuclear DNA fragments via vesicles that leads to anucleated but functional cytoplasts with phagocytic activity [8]. An interesting feature in mtDNA-based traps is the possibility that they could represent a molecular echo of mitochondria’s bacterial ancestry through endosymbiosis [9]. Remarkably, under quorum sensing signals bacteria are known to release extracellular DNA in both a lytic and a non-lytic manner, which is essential for biofilm formation, stabilization and integrity [10]. Notably, one critical role of biofilms is to shield bacteria from host immune defences, and one mechanism also involves the activation of pattern recognition receptors (PRRs) by extracellular DNA [11]. It is therefore tempting to speculate that mitochondrial DNA release in mammalian cells might represent a vestigial, evolutionarily conserved mechanism serving similar protective or intercellular communication roles. In line with that, mtDNA, which also harbours remnants of bacterial nucleic acid sequences, is a potent danger signal that can activate innate immune cells and modulate inflammatory responses [12].

A general concept that remains to be determined in NETosis is understanding which immune cell populations can extrude traps, whether they do so preferentially through vital or lytic NETosis, and for what purpose. Yousefi et al. proposed the coexistence of both mechanisms, early vital NET formation and late non-vital NETosis, within the same cells but occurring at different time points. They suggested that vital NET release, which takes place within just 5 minutes of stimulation, may represent an ultra-rapid mechanism to activate the immune system in response to infection [13].

From an evolutionary perspective, it is plausible that different types of DNA extrusion have evolved in distinct neutrophil subpopulations. One may hypothesize that less differentiated neutrophils, which retain a certain degree of plasticity, such as the so-called low-density neutrophils, might employ more conservative mechanisms compared to terminally differentiated neutrophils (mature normal density neutrophils).

In line, although initially observed in eosinophils and granulocytes stimulated with IL5+IFNg or GM-CSF and C5a, respectively, vital NET extrusion appears to be more likely in cells with a higher mitochondrial content [13]. A 2004 study demonstrated mitochondrial reshaping in neutrophils, highlighting their confinement to pro-apoptotic roles rather than ATP production, as well as their lower abundance compared to other PBMCs [14]. In this context, it would be interesting to investigate the extent of this mitochondrial reshaping during neutrophil differentiation, as well as the differences between normal-density and low-density neutrophils. The latter, according to their immature phenotype, might retain the ability to extrude mtDNA-based traps, potentially due to differences in mitochondrial composition. Still debated is also the mechanism supporting vital NET extrusion that can involve or not NADPH. The activation of NADPH oxidase is a hallmark of non-vital NETosis, a process that can involve the activation of different kinases including c-Raf-MEK-ERK and PKC [15] and that relies on autophagy, ROS and PAD4-dependent histone citrullination [16]. In addition to neutrophils, also monocytes, mast cells, T and B lymphocytes, and eosinophils have been reported to extrude extracellular traps [17]. In line with the above concepts, vital mtDNA-based traps are preferentially extruded by viable T-helper lymphocytes, serving as costimulatory signals for other T cells [18].

## The beneficial role of NETs

### Antimicrobial functions

It is well established that NETs are active players in the organism’s defence system. Their primary beneficial role lies in their ability to directly fight microbes through their specialized components. As previously mentioned, these extracellular traps are decorated with a variety of proteins, each with distinct antimicrobial functions. The sticky, web-like DNA filaments form a structural scaffold that captures invading pathogens, and through the removal of surface-bound cations they disrupt and lyse the bacterial membrane. The extrusion of the DNA trap is also beneficial to limit the spread of microbes to other tissues [19].

DNA-embedded proteins mediate antimicrobial activity through multiple distinct mechanisms. Neutrophil elastase (NE) exerts its function by degrading bacterial surface proteins and extracellular matrix components, impairing microbial virulence [20]. Similarly, MPO produces reactive oxygen species (ROS) and hypochlorous acid, which are toxic to bacteria [21]. Also, histones play a key defensive role. As fundamental components of NETs, they exert antimicrobial activity by directly binding to and neutralizing bacteria, promoting their elimination. These molecules can disrupt bacterial membranes, leading to bacterial lysis, and they further enhance the immune response by activating phagocytic cells and promoting the release of reactive oxygen species (ROS) from neutrophils, which further aid in pathogen clearance [22]. Furthermore, lactoferrin binds free iron, depriving bacteria of this essential nutrient and inhibiting their growth [23]. Likewise, gelatinase degrades bacterial proteins and extracellular matrix components [24]. Finally, calprotectin sequesters zinc and manganese, essential metals for bacterial growth, thereby inhibiting microbial proliferation [25].

### Immune adjuvant functions

Beyond their direct pathogen-killing function, NETs contribute to host defence by actively promoting and enhancing the immune response. Nuclear DNA-traps extruded by neutrophils in the extracellular space are engulfed by dendritic cells (DCs), endowed with antigen-presenting functions, and stimulated by the presence of natural immune adjuvants in the NET threads [26]. Indeed, the bulk of NETs is represented by chromatin which is composed of double-strand DNA (dsDNA) decorated with histones (H2, H3, H4). However, the presence of histones bonded to DNA, endows NETting cells with a double-edged sword power. Beyond their direct killing activity against bacteria and pathogens, NETs have been shown to kill mammalian cells with high efficiency, through mechanisms not completely understood, but that might include the triggering of Toll-like receptor (TLR) 2 and TLR4 by histones [27]. Accordingly, administration of antibodies against H3Cit or of PAD4 inhibitors can improve the prognosis of systemic inflammation and reduce tissue damage [28]. Such histone-mediated cytotoxic activity could also explain the toxicity of NETs during sepsis or the endothelial damage associated to some autoimmune diseases, such as autoimmune vasculitis (AAV). In a murine model of AAV, NETs obtained from neutrophils primed with anti-neutrophil cytoplasmic antibodies (ANCAs) promote endothelial cell (EC) damage in vitro, an effect that was prevented by DNase treatment, which degrades NETs. The outlined mechanism involves the activation of the alternative complement pathway with C3d fragment deposition on NETs and consequent C5a activation [29]. Notably, other than being involved in NET effector functions, molecules belonging to the complement cascade have been implicated in NET induction at distinct levels [30]. As an example, the activation of complement leads to the deposition of C3b and the generation of C5a. The latter has potent biological effects that amplify inflammatory responses. C5a is a chemoattractant for neutrophils, eosinophils, monocytes, and T lymphocytes, contributing to their recruitment to inflammatory sites [31]. Also, C5a when used in combination with interferons can directly stimulate NET extrusion [32]. Intriguingly, C1q supports NETs through the inhibition of DNase I activity, preserving extracellular DNA from degradation. However, histones are not the only effectors of this system, and chromatin itself can stimulate TLR9 to activate DCs when complexed with anti-microbial proteins, such as LL37 and high mobility group box 1 (HMGB1) [33–35]. Furthermore, dsDNA, which is the backbone of NETs, directly stimulates the cGAS/STING pathway, involved in various inflammatory diseases. cGAS stimulated by dsDNA converts ATP and GTP into cyclic GMP-AMP which activates STING to translocate into the Golgi apparatus, where it is phosphorylated to finally induce type I interferons. It has been shown that macrophages and myeloid cells are able to phagocytose NETs and consequently their dsDNA could be sensed by cGAS that, in turn, activates STING pathway leading to inflammation [36]. Similarly, mtDNA, in line with the evolutionary theory stated above, when released into the cytosol can activate immune pathways, such as cGAS/STING and inflammasomes (NLRP3), driving inflammation [37, 38]. Similarly, mitochondrial NETosis is triggered to trap pathogens but also elicit immune responses. However, both processes involve ROS production and can contribute to chronic inflammation and autoimmunity when dysregulated, as in SLE disease. In this regard, a study highlights the immune-adjuvant role of mitochondrial NETs, although it does not attribute antimicrobial activities to them, which are considered an exclusive function of nuclear NETs [39]. Overall, these observations support the idea that extracellular traps take part in the regulation of immune responses. In this context, we have contributed to demonstrating that extracellular traps have adjuvant properties, activating dendritic cells to present NET-associated antigens and thereby breaking self-tolerance and promoting autoimmunity [26]. Notably, to fully unleash the immunogenic properties of traps, their defective engulfment by macrophages seems necessary, as the elimination of traps by macrophages has been shown to maintain tolerance [40].

## NETs in cancer

### Tumour initiation, progression and recurrence

Although many components of NETs can stimulate the immune system, accumulating evidence indicates that NETs can be localized in the tumour microenvironment often playing a pro-tumoral role. Many types of cancer are linked to chronic inflammatory conditions, which often involve dysregulated NET production, a process that may contribute to tumour development, e.g. by fostering inflammation and genotoxic stress [41, 42]. In this regard, an interesting example is represented by chronic obstructive pulmonary disease (COPD), a predisposing factor for lung cancer [43]. In COPD, cigarette smoke has been shown to sustain NET formation, potentially amplifying an already active process. The COPD lung environment is particularly prone to infections, a hallmark of its pathogenesis, and is further shaped by a distinct microbial composition. This heightened NET activity may, in turn, drive tissue remodelling and pathological transformation. Interestingly, erythromycin, an antibiotic used to treat a variety of bacterial infections, can inhibit smoke-induced NETs [43], whether this effect could be endowed with a potential anti-tumoral effect is completely unknown and remains to be explored. Interestingly, excessive ROS production during neutrophil activation can be directly genotoxic but also it can reinforce NETosis by triggering extensive DNA damage and activating DNA repair pathways that ultimately lead to chromatin decondensation [44]. On the same line, MPO, which is released by neutrophils through NETs, can promote neoplastic transformation by producing genotoxic molecules like hypochlorous acid formed by MPO itself [45].

In the TME, NET formation is driven by a complex array of tumour-derived factors, including G-CSF [46], IL-8 [47] and TGF-β [48], as well as extracellular vesicles [49] released by cancer cells. Moreover, local conditions typical of the TME, such as hypoxia [50] and tissue stress (e.g. post-surgical inflammation) [51] further amplify NETosis. In addition, the presence of NETs themselves can stimulate the release of additional NETs in a positive feedback loop, largely mediated by NET components such as HMGB1, which is released during NETosis and acts via TLR4 and TLR9 signaling [52, 53]. Together, these inputs orchestrate a permissive and pro-inflammatory environment that facilitates neutrophil recruitment, chromatin decondensation, and the release of NETs. These stimuli can reprogram neutrophils toward a pro-tumoral phenotype prone to NETosis, further amplifying inflammation and immune evasion.

Other than cancer initiation, NETs contribute to tumour growth and dissemination. For example, NETs activate pancreatic stellate cells, leading to their proliferation and protease secretion, supporting primary tumour growth [54]. In metastatic colorectal cancer models, NETs enhance tumour growth by releasing neutrophil elastase, which activates TLR4 on cancer cells, triggering the p38–PGC1α pathway and promoting mitochondrial biogenesis, increased ATP production, and metabolic support for proliferation [53]. NETs may also stimulate cancer cell migration and invasion, acting as chemotactic factors through DNA sensing mechanisms and inducing epithelial-to-mesenchymal transition (EMT) [55]. Furthermore, neutrophils or NET components can either hinder or promote vascular remodelling, depending on the context, affecting the blood supply and therefore tumour size [56, 57]. Additionally, NETs play a crucial role in guiding cancer cells through the circulation, facilitating extravasation and establishment of the metastatic niche, either by themselves or through interactions with other players, such as platelets [58, 59]. In ovarian cancer NET entrapment within the vasculature enhances metastasis and NETs in premetastatic tissues support colonization of disseminating cancer cells [60]. One of the first pieces of evidence of NET involvement in cancer dates back to a few years ago, from the study of Egeblad’s group, which showed that the 4T1 metastatic breast cancer model induced the formation of NETs by neutrophils. Through elegant experiments, they demonstrated that NETs promote cancer cell migration and invasion in vitro, sustaining lung metastatic dissemination in vivo. Blocking NETs, either through DNase I-coated nanoparticles or PAD4 inhibitors, reduced metastatic burden in vivo [61]. Several detrimental activities of NETs within the TME are mediated by NET-associated proteases, such as proteinase 3 (PR3), neutrophil elastase, and matrix metalloproteinase 9 (MMP9), which can degrade extracellular matrix (ECM) components, thereby facilitating cancer cell proliferation and metastasis[62]. NE enhances tumour cell survival and migration through activation of the Src/PI3K/Akt signalling pathway, making it a potential target for therapeutic intervention [63]. Cancer recurrence is a major challenge in oncology and many efforts have been made to reduce its occurrence and to identify biomarkers of early relapse. Disseminated cancer cells may lie dormant until they are reactivated by external stimuli. NETs have been shown to play a role in this context by awakening dormant breast cancer cells through NET-derived proteases such as NE and MMP9, cleaving laminin and activating signalling pathways that resume cancer cell proliferation [64].

Also, redox imbalance and tumour-derived cytokines contribute to tumour-induced NET formation, further promoting metastasis [65]. Recently, it has been shown that chronic psychological stress can interfere with NETs formation, particularly via activation of glucocorticoid signalling in neutrophils, thereby supporting lung colonization in breast cancer models [66].

The pleiotropic effects of NETs in the TME also extend to T cells, compromising their anti-tumour activity. Specifically, NETs can hinder CD8⁺ T cell infiltration into tumours by creating dense physical barriers [67] and by downregulating T cell–recruiting chemokines such as CCL3 and CXCL9 [68]. In parallel, NETs suppress T cell activation by reducing the expression of key stimulatory molecules including CD80 and CD86 on dendritic cells and other antigen-presenting cells [69]. Furthermore, NETs can support regulatory T cell infiltration by inducing CD73 expression on tumour cells or by promoting IL-10 secretion in innate B cells [70, 71].

### Cancer comorbidity and organ dysfunction

Organ dysfunction is a common complication in cancer patients, with impaired kidney and heart functions being of particular concern. Among the mechanisms contributing to this dysfunction, NETs play a central role.

In kidneys, they act by obstructing the vasculature, leading to impaired perfusion and inflammation, a role further confirmed by the observation of restored renal function via inhibition of NETs in mice [72, 73]. Similarly, NET-associated cancer inflammation and myocardial stress can cause heart dysfunction in cancer patients [74]. This detrimental effect is further exacerbated by cardiotoxic chemotherapies, such as doxorubicin, where NETs actively contribute to cardiac damage [75]. Beyond vital organs, NETs are also involved in peripheral tissue injury, promoting chronic wounds and delayed healing, as seen in diabetic individuals [76] and they take part in thromboembolic events, which stand out as one of the most studied cancer-associated comorbidities, across tumour types [76]. The most common type of cancer-associated thrombosis is venous thromboembolism, which carries a nine-fold increased risk in cancer patients compared to the general population [77].

Cancer survivors have an increased risk of cardiovascular disease (CVD) and up to 50% of cancer patients exhibit histological evidence of venous thromboembolism, with some tumour histotypes being more exposed to this risk. A large epidemiological study has recently highlighted that the link between lung cancer and CVD is bidirectional, showing that CVD patients have an increased risk of developing NSCLC and that NSCLC patients have an increased risk of developing CVD [78], suggesting the existence of common pathogenic causes or commonalities in factors able to predispose or worsen the development of both diseases. This is something that has been suggested in the CANTOS trial in which the treatment with canakinumab, a humanized anti-IL-1β blocking antibody, reduced the risk of developing lung cancer up to 70% in patients receiving the higher doses of canakinumab [79]. Interestingly, there is a close link between IL-1β and NETs, which play relevant roles in CVD pathogenesis. In atherosclerosis that sets the stage for fatal thrombosis events, IL-1β contributes to the recruitment of neutrophils to atherosclerotic lesions that subsequently undergo NETosis. Interestingly, Warnatsch et al have shown that cholesterol crystals in atherosclerotic plaques can induce NET formation which in turn primes macrophages for IL-1β production thus promoting a self-amplifying IL-1β–IL-17 loop that sustains inflammation in the atherosclerotic plaque [80]. In the coagulation cascade NETs bind and activate the coagulation factor XII (FXII), and platelets [81] enhancing thrombin generation [82] and also stabilizing thrombi [83].

The interplay between NETs and tumour-driven hypercoagulability is increasingly recognized as a vicious cycle:

besides clinical factors (chemotherapeutics, immobilization), tumour-cell-specific procoagulant pathways and host immune responses can trigger thrombosis. An elevated platelet count has been associated with an increased risk of thrombosis in cancer patients [84]. Platelets are key mediators in this loop: they induce NET release through different factors such as the high mobility group box 1 protein (HMGB1), P-selectin, the α_IIb_β_3_ integrin complex, and choline transporter-like protein 2 (α_IIb_β_3_-CTL2) or direct neutrophil-platelet binding via glycoprotein Ib (GPIb) [85–89]. Conversely, NETs activate platelets through TLR2 and TLR4 [90] promoting their aggregation and coagulation. This reciprocal activation contributes to the heightened thrombotic risk observed in cancer patients. Consistently, elevated levels of NET-related biomarkers, such as H3Cit, nucleosomes, and cell-free DNA, are associated with a significantly increased risk of venous thromboembolism. In metastatic breast cancer NETs enhance procoagulant activity and predict poor patient prognosis [91]. Furthermore, in a prospective cohort of 946 individuals with newly diagnosed or progressive cancer, high H3Cit levels correlated with a significantly higher 2-year cumulative incidence of venous thromboembolism (14.5% vs. 8.5%), independent of tumour type and other established risks [92]. The close link between platelets, coagulation, and NETs was particularly evident in COVID-19 infection in which it has been shown that thrombotic events, which are common in the course of COVID-19, are related to the release of neutrophil extracellular traps (NETs) and mostly associated with patients with more severe outcomes [93]. Notably, both suicidal and vital NETosis were reported, the latter triggered by an IL-8–rich and the pro-oxidant environments, while vital NETosis also occurred in the case of concomitant bacterial triggering of TLR pathways [93].

Finally, other common cancer-related comorbidities, such as obesity, metabolic syndrome, and type 2 diabetes, can further potentiate NET formation [94, 95] thereby amplifying the prothrombotic and organ-damaging effects of NETs and intensifying the clinical burden of cancer-related systemic dysfunction.

So far, we have discussed about the involvement of NETs in organ damage. However, whether in turn NET-induced organ damage directly causes cancer is largely unknown. The most convincing association is between NET, fibrosis, and subsequent transformation. In a non-alcoholic steatohepatitis model, NETs promoted liver tumorigenesis through chronic inflammation and fibrosis, while NET inhibition reduced inflammation and tumour progression without affecting steatohepatitis development [96].

### NETs in anti-cancer treatment

Therapy resistance represents another major obstacle in cancer treatment, encompassing primary and acquired resistance to various therapies, including targeted therapies and immunotherapies. NETs impact therapy resistance at multiple levels, thus the possibility of targeting NETs presents an exciting avenue for future research and potential clinical trials, offering new opportunities to improve cancer therapy outcomes and patient prognosis. Although few studies have explored the clinical association between NET formation and chemotherapy response, initial in vitro and in vivo data suggest that NETosis contributes to chemoresistance. In multiple myeloma, neutrophils and PMN-MDSCs contribute to chemoresistance by releasing soluble mediators, including IL-6, IL-8, VEGF, and possibly ROS, that protect malignant plasma cells from doxorubicin-induced apoptosis while preserving their proliferative capacity. This protective mechanism, independent of direct cell–cell contact, suggests a functional reprogramming of the bone marrow microenvironment whereby neutrophil-derived factors buffer oxidative and cytotoxic stress, thereby mimicking the detoxifying role of NET-associated components and sustaining tumour cell survival under chemotherapeutic pressure [97]. A role of NETs in chemotherapy resistance has also been described in the 4T1 mouse mammary tumour model [64]. Chemotherapies induce NET formation and NET inhibition restores therapeutic activity. Mechanistically, cancer cells exposed to chemotherapy, release IL-1β that triggers NET formation, along with integrin αvβ1 and MMP9 that, associated with NETs, unleash and activate latent TGFβ [98]. Then, TGFβ induces tumour cells to undergo EMT, a process known to reduce cell proliferation and susceptibility to drugs acting on cycling cells [99].

Recent research indicates that neutrophils play a functional role in radiotherapy resistance as well, either alone or through NET formation, and their inhibition may represent a promising avenue to address post-radiotherapy resistance in clinics [100, 101].

In recent years, cancer immunotherapy, particularly immune checkpoint inhibitors (ICIs), has gained significant attention. The success of immunotherapy is influenced by the TME composition; however, the molecular mechanisms underlying ICIs resistance remain poorly understood. Recent studies have identified NETs as novel player in ICI resistance in certain tumours. NETs have been implicated in regulating tumour immunity by affecting immune cell function. Growing tumours establish a tolerant microenvironment, in which multiple immune suppressive mechanisms operate. Among them, MDSCs play a key part in several tumour types. One of the molecules responsible for their suppressive activity is arginase I (ARG1). This molecule is displayed on NET treads of patients affected by pancreatic ductal adenocarcinoma (PDAC) [102]. Consistently, a recent study by Canè and colleagues shows that, in PDAC patients, NETs from peripheral blood neutrophils are enriched in specific forms of ARG1, cleaved by cathepsin S and that these structures are endowed with enhanced enzymatic activity and contribute to T cell immune suppression [103]. While these cleaved ARG1 forms are not susceptible to ARG1 inhibitors, anti-ARG1 mAb restrains their activity. In a humanized mouse model, they tested, and demonstrated, in a melanoma in vivo model the efficacy of anti-ARG1 mAb in increasing the therapeutic activity of immunotherapies, either based on immune checkpoint inhibitors or ACT with T cells specific for a hTERT peptide [103]. While opening new windows for potential therapeutic approaches, this study also raises some issues regarding the use of small molecule ARG1 inhibitors that are being evaluated in phase I clinical trials.

In various cancer types, NET density inversely correlates with CD8 + T cell density, suggesting that NETs may restrain CD8 + T cell-mediated anti-tumour immunity [104]. Additionally, both CD4+ and CD8 + T cells in a NET-rich TME express higher levels of exhaustion-related markers. It has been shown that NETs indeed contain immunosuppressive ligands like PD-L1, which may contribute to TME immunosuppression and tumour outgrowth [105]. Furthermore, NETs can promote the differentiation of naïve CD4 + T cells into regulatory T cells (Tregs) in certain cancer types [106]. On the same line of evidence, elevated levels of NETs have been associated with decreased responsiveness to anti-PD-1 and cytotoxic T-lymphocyte associated protein 4 (CTLA4) therapies, further emphasizing their impact on immunotherapy outcomes [107]. Accordingly, inhibition of NETs has been shown to sensitize tumours to PD-1 checkpoint blockade [108] opening new possibilities to overcome immunotherapy resistance.

While the prevailing view, supported by this growing body of evidence, is that a high neutrophil-to-lymphocyte ratio (NLR) and the presence of NETs are associated with reduced efficacy of immunotherapy and poorer clinical outcomes, it is important to acknowledge, that a few studies have reported opposing results. Although limited in number, these studies suggest that neutrophils and NETs may, in certain contexts, contribute to the establishment of an effective and durable anti-tumour response following immunotherapy. In particular, two recent studies shed light on this crucial point. Hirschhorn and colleagues discovered that neutrophils are vital for overcoming antigen escape in melanoma when treated with CD4 + T cell therapy, combined with OX40 co-stimulation or CTLA4 blockade [109]. This combination not only activates neutrophils but also triggers an anti-tumour response through a nitric oxide synthase–dependent mechanism. In parallel, Gungabeesoon and colleagues found that in lung cancer models, successful immunotherapy triggers a rapid expansion of tumour-infiltrating neutrophils, which express a key IFN gene signature necessary for tumour control [110]. In their study, they demonstrated that this kind of response is fuelled by BATF3+ dendritic cells, IL-12, IFNγ, and CXCR3. Moreover, they also showed that in human lung cancer patients, a strong neutrophil response post-treatment was linked to improved outcomes [110].

## The paradox: tolerance versus immune activation

In view of such observations, a relevant question is why NETs are not providing immune adjuvant activities in the TME. The majority of studies, including our current research on lung cancer and lymphoma patients, have highlighted that different tumour contexts, not only those arising from autoimmune conditions that might in some way foster NET extrusion, are characterized by excessive NETosis and malignant transformation [111]. As described above, higher NETosis causes inflammation and tissue damage, resulting in cancer initiation, progression, recurrence, comorbidities and resistance to therapy. As highlighted in the review by Tan et al., there is still debate on whether NETs actively initiate inflammation and tissue damage, or if they are instead a consequence of ongoing inflammation that perpetuates further injury [112]. Nonetheless, the biology of this type of NETosis must be different from the NETosis occurring in healthy contexts, somehow attributing to cancer-related NETs an immune suppressive role [113]. For instance, the TME is poor in cells differentiated towards antigen presentation in favour of tolerogenic cells [114]. In this context, NETs could contribute to the so-called low-grade smouldering inflammation, which promotes tumorigenesis, while supporting tolerogenic mechanisms [115].

Another possibility that might explain the immune suppressive activity of NETs in the TME is the preferential pro-tumorigenic N2 polarization, rather than anti-tumour N1, of cells that are actively extruding traps. In line, cancer patients display an increased amount of circulating low density N2-like neutrophils and they may consequently produce immune suppressive NETs [116].

In line with the possibility that neutrophil polarization and differentiation may have a role in tumour immune response, Yam et al. have shown that tumour neutrophils convert from a wound-healing phenotype to a cytotoxic anti-tumour phenotype upon exposure to microbial bioparticles of *Mycobacterium bovis* Bacillus Calmette Guerin (BCG) [117]. The anti-tumour activity of BCG-primed neutrophils was associated with their ability to recruit cytotoxic CD8 T cells, thus explaining the synergistic effect of BCG with immunotherapy (a concept we will revisit later). The link between neutrophils and CD8 T cells is not completely new, as shown in an old study in which a murine colon carcinoma transduced with G-CSF induced strong neutrophil recruitment, leading to CD8-mediated tumour rejection [118]. In this study, neutrophils recruited by implanting an agar pad subcutaneously in tumour-bearing mice exhibited a cytostatic effect on the tumour, paralleled by an increased capacity to extrude traps, as demonstrated in subsequent studies [26].This might imply that either de novo recruitment of neutrophils and their commitment to anti-tumour functions, or a redirection of pre-existing immunity, could be beneficial for ICI effectiveness. Supporting the notion that neutrophil polarization toward an N1 phenotype can modify the impact of NETosis on tumour cells, Liu and colleagues showed that BCG-induced NETs exert cytotoxic effects on bladder cancer cells. This occurs through the release of pro-inflammatory cytokines and recruitment of T cells and monocytes, ultimately inducing apoptosis and cell cycle arrest [119]. Aside from a study in melanoma demonstrating a direct cytotoxic effect of NETs on tumour cells [120] their presence in vivo appears to influence neutrophil anti-tumour activity indirectly, such as through cytotoxic effects on the endothelium [121]. Notably, the study by Schedel et al. provides compelling evidence of NETs in patient biopsies, localizing them specifically within the ulcerated regions of melanoma lesions [120]. This might reflect an underlying vascular damage, potentially reducing oxygen supply, which could be ultimately dependent on the cytotoxic effect of NETs over ECs [29], an effect that is in part mediated by extracellular histones and that relies on the specific expression by activated ECs, of adhesion molecules such as P-selectin, which, on one hand supports EC adhesion and, on the other, is a potent inducer of NET formation, by engaging PSGL-1 counter-ligand on neutrophils [122, 123].

While most of these studies highlight the role of NETs within the complex TME, a few have addressed whether NETs themselves, independently of other immune components, can exert direct cytotoxic effects on tumour cells. To specifically assess the direct effects of NETs on cancer cells without interference from cellular interactions, Arelaki et al. used NETs isolated from neutrophils of healthy donors stimulated in vitro with PMA or sepsis serum and applied these purified structures, in the absence of intact neutrophils, to cultures of colorectal cancer and primary AML cells [124].This approach allowed them to exclude any contribution from live neutrophils or endothelial interactions, isolating the intrinsic cytotoxic properties of NETs. Both PMA- and sepsis-induced NETs triggered apoptosis in both cell lines, while in AML cells also reduced proliferation. These effects were abolished by treatment with DNase or heparin, indicating that the cytotoxic and anti-proliferative activity of NETs depends on the integrity of their chromatin scaffold. Whether NETs generated in response to cancer cells differ functionally or structurally from those induced by infectious stimuli remains to be explored. The exact mechanisms by which NETs recognize and kill human cancer cells, possibly via histones, proteases, or receptor-mediated pathways, also remain incompletely understood.

**Fig. 2:**
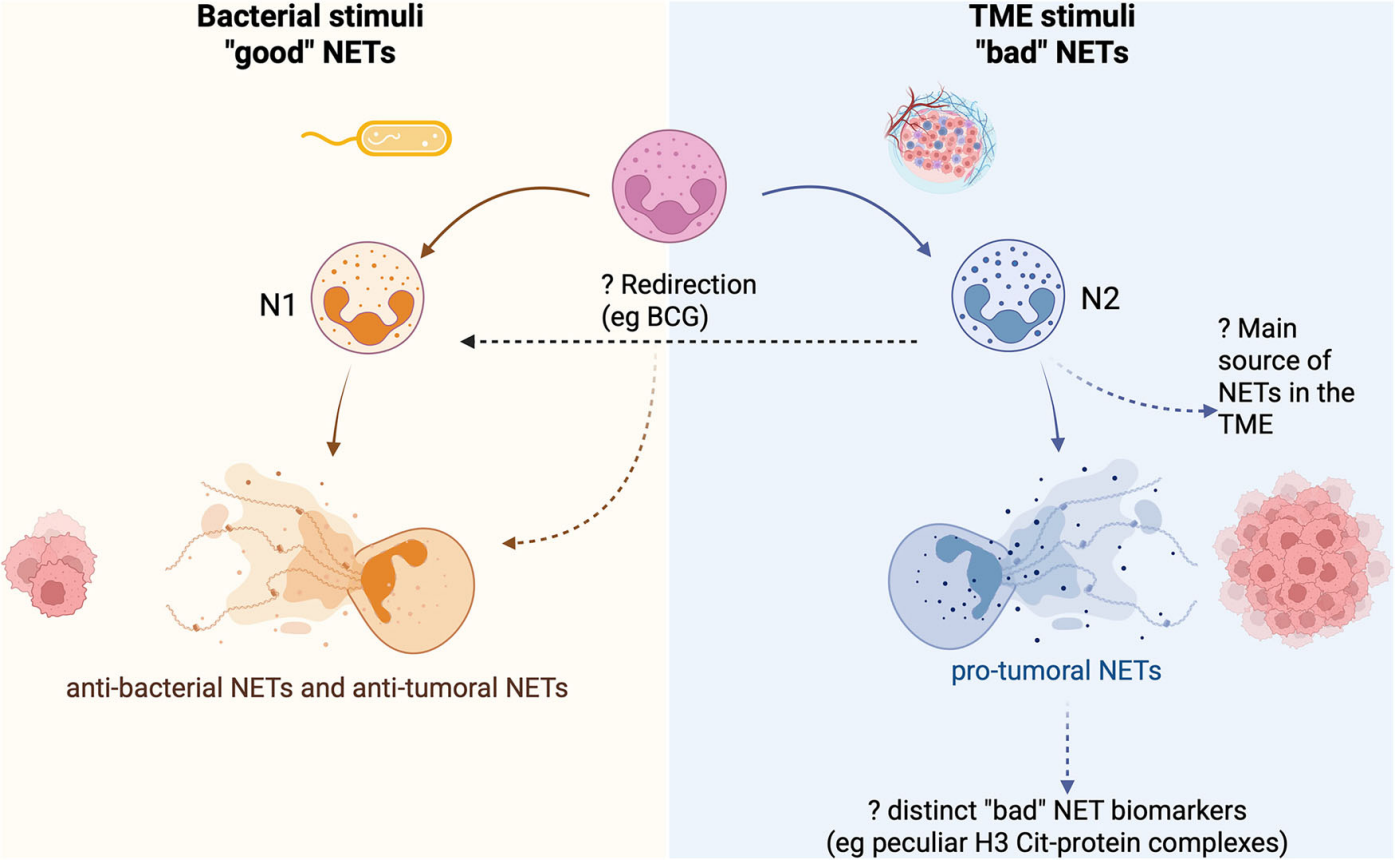
Context-dependent roles of NETs in tumour biology. Under bacterial stimulation, neutrophils are primed to induce an antimicrobial response, which has been demonstrated to exhibit anti-tumour effects when antigens are shared between tumour cells and bacteria. Conversely, the TME fosters the activity of N2 neutrophils, which support tumour growth, partly through the formation of NETs that promote immune suppression and tolerance. Dotted arrows indicate some open questions: whether "bad" NETs can be redirected towards anti-tumour functions; what is the main source of NETs in the TME; and the current lack of relevant markers for “bad” NETs.

Intriguingly, it has been suggested that microbiota-derived components and tumour cells may share specific epitopes presented by HLA-restricted antigens, thereby redirecting CD8⁺ memory T cells, originally primed by microbial antigens, to recognize and attack tumour cells [112]. Whether this antigenic homology, or the local expression of bacterial components by tumour cells infected in situ [113] involves molecules also recognized and targeted by NETs remains an open and fascinating question. Interestingly, some of the most frequent tumour-associated antigens sharing homology with microbe-derived ones are MAGEs [125] that are highly expressed in melanoma, which represent one of the few tumour contexts in which NETs have been shown to directly lyse tumour cells [120]. Indeed, digital spatial profiling and single cell RNAseq analyses conducted by Galeano Niño and colleagues on oral squamous cell and colorectal cancers have shown that some tumour areas are enriched in infiltrating immune suppressive cells and, interestingly, these areas are also populated by bacteria. Such bacteria infect neoplastic cells, resulting in their genetic alteration that finally leads to MDSC recruitment [126]. Taking together all these pieces of evidence, it could be postulated that when NETosis is triggered by bacterial stimuli, resulting in an antibacterial phenotype in line with its normal function, it can also inhibit cancer cell growth perhaps in the presence of bacterial-tumour antigen mimicry (Fig. 2). Whether these different phenotypes are more prone to vital or suicidal NETosis is still under exploration.

## Perspective: is it time to consider NET-targeting treatments?

Overall, the presence of NETs in the tumour microenvironment poses a formidable challenge to cancer treatment. Consequently, there is a growing interest in targeting NETs to restore immune sensitivity and improve the success of immunotherapeutic interventions. However, the dual role of NETs, as both immunosuppressive agents and components of the host’s antimicrobial defence, introduces a critical therapeutic dilemma. Moreover, the presence of NETs during anti-tumour neutrophil responses suggests some notes of caution.

Chamardani and Amiritavassoli, in their review, have listed the most important NET inhibitors [127]. Among them, a promising approach to overcome NET-mediated resistance/tolerance is NET degradation using DNase I. This endonuclease can break down the DNA backbone of NETs, disrupting their structure and neutralizing their immunosuppressive effects. Preclinical studies have shown that treating tumour-bearing mice with DNase I enhances the efficacy of ICIs. By dismantling the NETs, DNase I facilitates the infiltration of CD8+ cytotoxic T cells into the tumour, allowing them to engage and destroy cancer cells more effectively [86]. In addition to DNase I, or alternatively, it may be possible to block NET formation by targeting specific components involved in their generation, such as PAD4, NE, and MPO, as a mean to restore immune response. Indeed, inhibition of PAD4 using GSK484 has been shown to decrease NET formation and suppress tumour growth in mouse models [128]. Also, preclinical studies using NE [129] or MPO [130] inhibitors have demonstrated their potential to reduce metastasis and inhibit NET-mediated immunosuppression in cancer. Tumour-associated factors involved in neutrophil recruitment and NETosis promotion, such as IL-8, IL-17, and their receptors, may be potential targets, since their blockade has already been shown to effectively inhibit cancer growth, while improving anti-tumour immunity [131].

While these strategies hold significant therapeutic promise, a major obstacle to their clinical translation is the potential disruption of innate immunity. Broad inhibition of NET formation, such as through DNase treatment or PAD4 inhibitors, may effectively target pro-tumoral NETs, but it is crucial to strike a balance between suppressing harmful NET activity and preserving those with protective antimicrobial and possibly anti-tumoral functions. NETs play a vital role in host defence against infections, and their indiscriminate inhibition could lead to serious complications, particularly in immunocompromised cancer patients [132, 133]. For example, in lung cancer patients, a setting where the tumour develops in a microenvironment highly reliant on neutrophil-mediated defence against airborne and circulating pathogens. This contrasts with organs such as the liver and spleen, where circulating pathogen clearance is predominantly mediated by macrophages. Therefore, systemic NET inhibition in lung cancer may carry a higher risk of compromising host defence mechanisms compared to other tumour types [134, 135].

In light of this, a key future direction will be to better define the tumour-specific mechanisms underlying NETosis induction and function. This could aid in identifying therapeutic targets that inhibit detrimental effects of NETs without compromising their beneficial immune-adjuvant roles.

This could be selectively achieved by identifying molecular or phenotypic features that distinguish “bad” NETs, those that suppress immunity and support tumour progression, from “good” NETs involved in antimicrobial defence and immune activation. An underestimated aspect of NETosis in cancer that deserves further investigation is the extent to which NETosis depends on altered neutrophil efferocytosis. This process might be associated with dysregulation of ‘eat-me’ and ‘don’t-eat-me’ signals, or with defective macrophage-mediated clearance [136] ultimately resulting in the persistence of “bad” neutrophils and their subsequent NETosis. While the stimuli that induce NETosis within the TME are well characterized, the possibility that these same stimuli may also impair neutrophil clearance remains largely unexplored. If this proves to be the case, drugs such as anti-CD47 antibodies [137] could find a novel therapeutic application in blocking cancer-associated detrimental NETosis. The identification of tumour-associated “bad” NETosis is crucial not only for treatment purposes but could also be exploited as a detection tool. So far, some efforts have been made to exploit the measurement of biomarkers such as citrullinated histones, cell-free DNA, NE, and nucleosomes. However, these markers are present on every type of NETs, being associated both with anti-microbic/autoimmunity and pro-tumoral NETs and are not able to differentiate these two opposite functions. Therefore, a more specific biomarker that may identify pro-tumoral NETs is needed for developing suitable treatment strategies. For example, the identification of specific complexes between citrullinated histones and immunosuppressive molecules (e.g arginase–histone complexes) could define a subclass of ‘bad’ NETs capable of both dampening anti-tumour immunity (treatment purpose) and signalling the presence of cancer (diagnostic purpose).

Finally, several aspects remain to be elucidated, including the distinct contributions of vital NET formation versus NETosis within the tumour microenvironment, as well as the role of mitochondrial-derived NETs. At present, there is a lack of concrete evidence regarding which release mode of NETs plays the dominant pro-tumorigenic role. To date, only one study in breast cancer has shown that vital NETs, formed independently of PAD4 activation and H3 Cit upregulation, are derived from aged neutrophils and observed in lung tissue, where they contribute to pre-metastatic niche formation and promote tumour progression [138]. Moreover, while mitochondrial NETs can be distinguished by assessing mitochondrial markers on the extruded DNA, discriminating vital NETs in vivo remains challenging. The enzymes involved and the DNA-associated markers are the same; what differs is the type of stimuli that trigger them. It is plausible that a microenvironment enriched in IL-8 may promote NETosis, and one may further speculate that the local tumour-associated microbiota could create niches capable of inducing the extrusion of vital NETs, a process that engages TLRs.

Another challenging aspect is that other cell types are also capable of extruding extracellular traps; therefore, identifying and quantifying the cellular sources of these traps within the tumour microenvironment represents another area that warrants further investigation. For instance, to what extent are citrullinated histones decorated with MPO or PR3 proteins within the tumour milieu? What is the relative contribution of each cellular source, and what roles do they play? These are all critical questions that deserve comprehensive exploration.
